# Effectiveness of Resistance Training of Masticatory Muscles for Patients With Temporomandibular Disorders: A Systematic Review

**DOI:** 10.1111/joor.14021

**Published:** 2025-05-25

**Authors:** Giacomo Asquini, Giulia Pisacane, Filippo Maselli, Firas Mourad, Paolo Bizzarri, Edoardo Balli, Cecilia Bagnoli, Anna Manzari, Marco Pernici, Andrea Giusti, Deborah Falla

**Affiliations:** ^1^ Centre of Precision Rehabilitation for Spinal Pain, School of Sport, Exercise and Rehabilitation Sciences, College of Life and Environmental Sciences University of Birmingham Birmingham UK; ^2^ Department of Human Neurosciences University of Roma “La Sapienza” Rome Italy; ^3^ Department of Health LUNEX University of Applied Sciences Differdange Luxembourg; ^4^ Luxembourg Health & Sport Sciences Research Institute A.S.B.l. Differdange Luxembourg; ^5^ Experimental Anatomy Research Group (EXAN) Vrije Universiteit Brussel (VUB) Brussels Belgium

**Keywords:** exercise, masticatory muscle, pain, physical therapy, resistance training, temporomandibular disorder, temporomandibular joint

## Abstract

**Background:**

Evidence supports the use of exercise for patients with temporomandibular disorders (TMDs). However, previous studies have mainly focused on combined treatment strategies or undefined exercise modalities.

**Objective:**

This systematic review aims to evaluate the effectiveness of Resistance Training (RT) as a standalone treatment for managing pain and improving neuromuscular performance in individuals with TMDs.

**Methods:**

This systematic review followed a pre‐established and published protocol, which was registered with PROSPERO (CRD42023476269). The literature search was conducted from March 1st 2024 to March 31st 2024 via the following electronic databases: MEDLINE (OVID interface), EMBASE (OVID interface), SCOPUS, Web of Science, PubMed, and Cochrane Central Register of Controlled Trials (CENTRAL). Randomised controlled trials or nonrandomised studies of interventions were included when they compared the effect of RT targeting masticatory muscles on pain, neuromuscular performance, and maximum mouth opening in patients with TMDs versus other treatment modalities. Two independent reviewers screened articles for inclusion, extracted data, assessed the risk of bias using the revised Cochrane risk of bias tool for randomised trials and evaluated the overall quality of evidence using the Grading of Recommendations, Assessment, Development and Evaluations approach.

**Results:**

From an initial 2177 articles, only three met the inclusion criteria and involved 108 participants. All the included studies demonstrated a decrease in pain intensity and an improvement in neuromuscular performance following RT, even if the superiority of RT over other interventions remains uncertain. However, the combination of moderate risk of bias, significant heterogeneity and small sample sizes resulted in a very low quality of evidence.

**Conclusions:**

Clinicians managing patients with TMDs should consider RT as an effective, conservative option in conjunction with other treatment modalities. Future methodologically robust studies with large sample sizes and clearly defined exercise protocols are needed to investigate the role of RT for reducing TMD‐related pain by increasing load tolerance and addressing potential bruxism‐related muscle overload.

## Graphical abstract

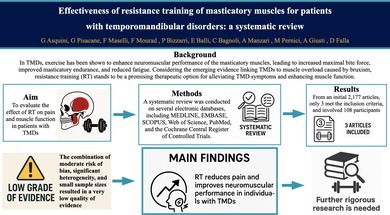

## 1 Background

Temporomandibular disorders (TMDs) are one of the most common musculoskeletal disorders in developed countries, surpassed only by chronic low back pain [1, 2]. TMDs represent the leading cause of non‐odontogenic orofacial pain and often persist chronically [3]. Approximately 15% of adults, particularly those aged 20 to 40, are affected by painful TMDs [4]. These conditions can greatly interfere with daily activities, emotional health and overall quality of life, while also leading to significant economic burdens from treatment costs and decreased work efficiency [2, 5, 6, 7]. TMDs are characterised by pain, joint sounds and restricted movement in the temporomandibular joints (TMJs), masticatory muscles and associated structures [2]. Patients may also exhibit altered neuromuscular function of the masticatory muscles, including reduced bite strength, muscle fatigue during sustained efforts and compromised oxygen extraction that impacts muscle performance [8, 9, 10, 11].

Clinical guidelines currently advocate for a multidisciplinary approach when managing patients with TMDs, incorporating conservative treatments such as education, manual therapy, exercise and oral appliances [12, 13, 14, 15, 16]. Recent research highlights the effectiveness of exercise in alleviating pain, enhancing TMJ range of motion (ROM) and improving overall function in individuals with TMDs [17, 18]. Strengthening exercises have proven beneficial for various musculoskeletal conditions, including chronic lower back pain and neck pain [19, 20, 21, 22]. Evidence indicates that resistance training (RT) can increase muscle strength, reduce pain and enhance functional capacity in patients with chronic low back pain, knee osteoarthritis, chronic tendinopathy and those undergoing recovery after hip replacement surgery [23]. Furthermore, RT plays a crucial role in maintaining muscle health, improving functional outcomes and mitigating inflammation by elevating anti‐inflammatory factors like interleukin‐10 and adiponectin while decreasing inflammatory markers such as C‐reactive protein and interleukin‐6 [24, 25]. In patients with TMD, exercise has also been shown to enhance neuromuscular performance of the masticatory muscles, leading to increased maximal bite force, improved masticatory endurance and reduced fatigue [26, 27]. Considering the emerging evidence linking TMDs to muscle overload caused by bruxism [28, 29], resistance training stands to be a promising therapeutic option for alleviating TMD symptoms and enhancing muscle function.

Previous systematic reviews have explored the effectiveness of exercise for managing TMDs, yet the findings remain contentious [17, 18, 30, 31, 32, 33]. For instance, in a review by Zhang et al. [32] there was no significant difference in pain reduction between exercise and splint therapy. In contrast, the review by Dickerson et al. [31] highlighted moderate short‐term improvements for pain and TMJ ROM following mobility exercises or combined treatment approaches. The review by Idáñez‐Robles et al. [33] indicated that exercise could alter the pressure pain threshold (PPT) and improve mouth opening, particularly when used alongside occlusal splints. A review by Shu et al. [18] noted enhancement of maximal bite force among adults performing oral exercises, and the review by Shimada et al. [17] affirmed the beneficial effects of exercise on pain intensity and jaw mobility in individuals with TMD classified as myalgia and arthralgia. Although Armijo‐Olivo's review [30] reported positive outcomes with both active and passive exercises for the jaw and neck, it did not find these interventions to be superior to other conservative treatments for patients with TMDs. Notably, many previous reviews included participants with various TMD types, such as myalgia, arthralgia and disc displacements [30, 31, 32, 34], or even healthy individuals [18]. Moreover, some investigations assessed combined treatment approaches that included exercises [33], while others focused on mixed modalities (e.g., mobilisation exercises paired with resistance training) [17, 18, 30] or utilised forms of exercise that lacked clear definition or description.

To the best of our knowledge, there has not been a systematic review of the evidence investigating the effectiveness of RT for the management of patients with TMDs. Thus, this systematic review aimed to fill that gap by evaluating the effect of RT on pain and muscle function in patients with TMDs.

## 2 Methods

### 2.1 Protocol and Registration

This systematic review was carried out in accordance with the guidelines set forth by the Preferred Reporting Items for Systematic Reviews and Meta‐analyses Protocols (PRISMA‐P) [35] (for additional details, see Additional File S1) and is registered with PROSPERO (CRD42023476269). The full systematic review protocol has also been published [36] and there were no deviations from the protocol.

### 2.2 Eligibility Criteria

#### 2.2.1 Inclusion Criteria

The selection criteria adhered to the PICOS framework (Population, Intervention, Control, Outcomes and Study Design) as recommended by the PRISMA checklist [35].

##### 2.2.1.1 Participants

Eligible participants included adults aged 18 years and older who had received a diagnosis of TMD based on the Research Diagnostic Criteria for TMD (RDC/TMD) or the Diagnostic Criteria for TMD (DC/TMD) [37, 38], or trials that included a population exhibiting signs and symptoms of a TMD [2].

##### 2.2.1.2 Intervention

Only trials examining exercise of masticatory muscles described as RT or strengthening exercises were considered eligible. For this review, RT was defined based on the PubMed medical subject headings as ‘a type of strength‐building exercise program that requires the body muscle to exert a force against some form of resistance, such as weight, stretch bands, water or immovable objects. Resistance exercise is a combination of static and dynamic contractions involving shortening and lengthening of skeletal muscles’. Trials that incorporated an educational component in addition to RT were also eligible.

##### 2.2.1.3 Study Design and Comparison

Eligible studies consisted of randomised controlled trials (RCTs) comparing RT alone to comparison groups that did not include RT. In cases where RCTs were unavailable, high‐quality non‐RCT studies were included as recommended in the Cochrane Handbook for Systematic Reviews [39]. Any type of comparison group was acceptable (e.g., standard care, splint therapy, sham interventions) as long as it did not include RT.

##### 2.2.1.4 Outcome Measures

The primary outcome was pain intensity. Only trials that evaluated pain using methodologies endorsed by the Initiative on Methods, Measurement and Pain Assessment in Clinical Trials (IMMPACT) [40] (e.g., visual analogue scale, numeric rating scale) were considered eligible. Pain was defined as pain localised to the TMJ area, masticatory muscles, or referred pain to associated structures. Secondary outcomes encompassed neuromuscular performance, TMJ ROM and PPTs over the masticatory muscles. Neuromuscular performance outcomes included masticatory muscle efficiency (such as endurance time and force generation during biting tasks) and maximum voluntary isometric contraction (MVIC). TMJ ROM was included, assessed by measuring the maximal mouth opening (MMO) via interincisal distance in millimetres during active MMO, in accordance with the DC/TMD physical examination guidelines [37].

##### 2.2.1.5 Timing and Setting

All follow‐up assessments conducted at any time were included, and there were no restrictions regarding the setting or duration of the intervention and follow‐up.

#### 2.2.2 Exclusion Criteria

Trials that examined the effects of RT in conjunction with other treatment modalities, excluding education (e.g., manual therapy, mobilisation exercises or similar interventions), were excluded. If it was unclear whether the RT intervention was focused solely on the masticatory muscles and included other muscle groups (e.g., neck muscles), the trial was not deemed eligible for inclusion in the systematic review. Additionally, studies involving participants with rheumatic diseases, neurological disorders, cancer, or those who had undergone surgery in the TMJ region were excluded. Papers not published in English were also excluded from this review.

### 2.3 Information Sources

The search for relevant studies was conducted from March 1 to March 31, 2024, across several electronic databases, including MEDLINE (via OVID), EMBASE (via OVID), SCOPUS, Web of Science, PubMed and the Cochrane Central Register of Controlled Trials (CENTRAL). To identify supplementary literature, reference lists of included studies and related systematic reviews were scrutinised. Additionally, hand searching was performed in journals focused on exercise and TMD topics, specifically the *Journal of Oral Rehabilitation*, *Musculoskeletal Science and Practice*, *Physical Therapy*, *Clinical Rehabilitation*, *The Journal of Oral & Facial Pain and Headache*, *Journal of Applied Oral Science* and *Journal of Dental Research*. Searches were also conducted on the British National Bibliography and EthOS to locate grey literature and unpublished research.

### 2.4 Search Strategy

The initial bibliographic search was executed in PubMed and subsequently modified for other databases. The search strategy employed medical subject headings (MeSH terms) when available, along with Boolean operators, truncation, wildcards, quotation marks and keywords related to TMD, TMJ, MMO, RT, exercise, physical therapy and pain. No date restrictions were imposed. Detailed search strategies for all databases are presented in Additional File S2.

### 2.5 Selection Process

**FIGURE 1:**
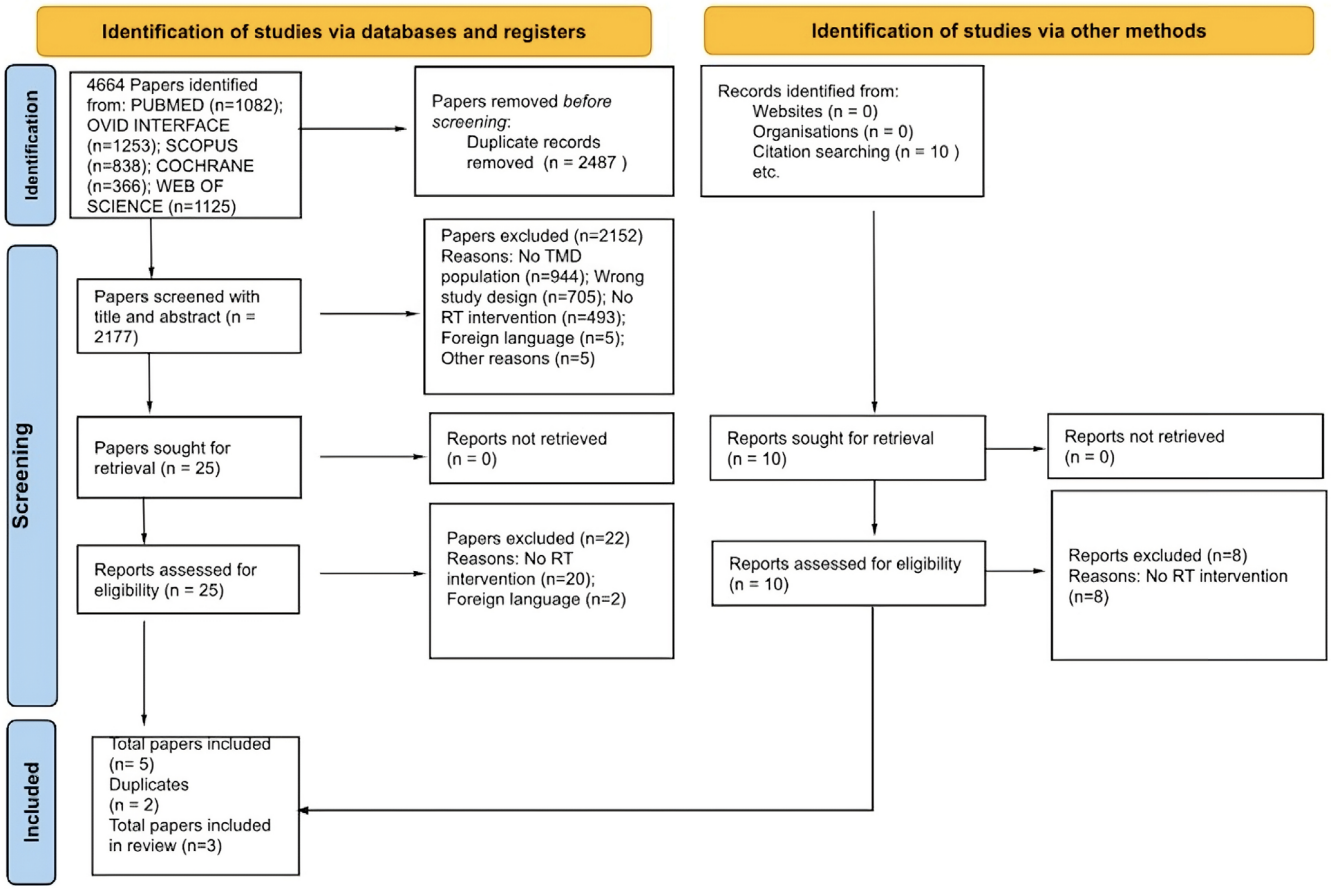
PRISMA flow diagram which included searches of databases, registers and other sources [35].

The selection of studies was carried out by two independent reviewers (GA/GP), who separately examined the titles and abstracts of all potentially eligible articles using the predefined criteria [41]. The eligibility criteria were prioritised in the following order: (1) participants, (2) study design, (3) type of intervention, (4) outcome measures and (5) absence of exclusion criteria. If a trial could not be excluded based solely on the title and abstract, the full text was reviewed. Only those papers that both reviewers deemed eligible were included. In the event of disagreement, the reviewers discussed the reasons for their differing opinions and sought to reach consensus. If necessary, a third reviewer (DF) was consulted for mediation. The level of agreement between reviewers is documented in Additional File S3, while the flow of inclusion and exclusion of articles is illustrated in a diagram (Figure 1), as recommended by PRISMA guidelines [35].

### 2.6 Data Collection Process

A tailored proforma, aligned with the Cochrane template [39], was developed and tested for extracting data from the included trials. Both reviewers (GA/GP) independently processed the information, consulting the third reviewer (DF) when discrepancies arose.

### 2.7 Data Items

Table 1 outlines the specific items extracted from the included studies. When necessary, authors were contacted for further details.

**TABLE 1:** Summary of items to be extracted from included trials.

Content	Data items
Trial information	Authors, year of publication, Country
Population	Number of participants, type of TMD, inclusion and exclusion criteria
Intervention	Protocol of exercises proposed
Comparison group	Intervention for the comparison group
Outcome measures	Type of outcomes considered in the trial and measurement tool
Follow‐up assessment points	Detail of timing for each group and outcome measured
Results	Differences in the outcomes between exercise group and comparison

### 2.8 Risk of Bias in Individual Studies

The risk of bias in the included studies was evaluated using the revised Cochrane risk‐of‐bias tool for randomised trials (RoB2) [42], which is regarded as the optimal method for assessing RCTs [43]. The risk of bias was assessed for each included study, with the independent reviewers (GA/GP) grading each domain according to the comprehensive guidance provided by the RoB2 Development Group [42]. Domain‐level judgements and overall assessments of risk of bias (low risk, some concerns and high risk) were determined based on responses to questions structured hierarchically by the RoB2 tool. In instances of disagreement, a third reviewer (DF) was brought in for resolution. Agreement levels among reviewers are presented in Additional File S4.

### 2.9 Synthesis Methods

A qualitative synthesis was conducted, where summary tables were used to present key details from each study, including TMD type, exercise dosage, comparators, outcomes and assessment time points. Due to the heterogeneity observed among the included studies regarding comparators, resistance training dosage (in both training protocols and force applied), outcomes and the scales used for their assessment, it was not feasible to calculate the standardised mean difference and the 95% confidence intervals. Consequently, only a narrative synthesis was conducted to report the results of the studies, and tabular methods were used for data presentation, as suggested by the ‘Synthesis without Meta‐Analysis’ (SWiM) guidelines [44]. The tables highlighted essential trial characteristics such as study design, sample size, comparators, time points and risk of bias (RoB2). No additional or sensitivity analyses were conducted, given the heterogeneity and limited number of included studies.

### 2.10 Reporting Biases Assessment

Unpublished studies were sought, alongside an evaluation of consistency between study protocols, trial registration and the published articles. Competing interests from various author groups were also assessed. No unpublished studies were identified, and no conflicting interests among authors were found. Two of the included studies did not have a registered protocol.

### 2.11 Certainty Assessment

The overall quality and strength of evidence were evaluated by two independent reviewers using the Grading of Recommendations, Assessment, Development and Evaluations (GRADE) framework [45]. This method assesses five key domains: risk of bias, inconsistency, indirectness, imprecision and publication bias. Based on these factors, the quality of evidence was rated from high to very low [45].

## 3 Results

### 3.1 Study Selection

Figure 1 outlines the trial selection process for this systematic review. Of the 2177 nonduplicate articles initially screened by title and abstract, 25 full‐text articles underwent further evaluation. Ultimately, three studies were included in the review [27, 46, 47]. Articles were excluded for various reasons, such as no sample population with TMD (*n* = 944), incorrect study design (*n* = 705), no RT intervention or RT alone (*n* = 493), being written in a non‐English language (*n* = 5) or other reasons (*n* = 5).

### 3.2 Study Characteristics

Table 2 details the three included studies conducted in Germany, Brazil and Israel, encompassing 108 participants and published between 2006 and 2019 [27, 46, 47]. The Research Diagnostic Criteria for TMD (RDC/TMD) were used in all studies, with significant systemic medical conditions as common exclusion criteria. All three studies exclusively enrolled women aged 18–45 years [27, 46, 47] and the inclusion of female participants only is worth noting. Heterogeneity was observed in the inclusion criteria. One study focused on chronic pain [46], another excluded those with higher impact chronic pain (scores 3–4 on the GCPS scale) [27] and the third required pain lasting at least 6 months [47]. Variations also existed in comparators (sham therapy, education/support, splints) and secondary outcomes, such as muscle efficiency, PPT and electromyography (EMG) measures.

**TABLE 2:** Characteristics of studies comparing RT vs. other interventions for improving pain intensity and MMO in TMD populations.

Trial info	Population	Intervention	Comparison	Outcome measure	Follow up	Results
Giannakopoulos et al. [27] Year: 2018 Country: Germany	‐**Sample**: 42 women aged between 18 and 45 year (Group A: 20 aged 28.2 ± 6.4 years, Group B: 22 aged 24.7 ± 3.4 years) with nonchronic (i.e., nondysfunctional) myofascial TMD ‐**TMD type**: myofascial TMD pain ‐**TMD diagnostic criteria**: German version of the Research Diagnostic Criteria for Temporomandibular Disorders (RDC/TMD). ‐**Inclusion criteria**: myogenous pain values of 1 or 3 according to the Graded chronic pain status (GCPS) ‐**Exclusion criteria**: pregnancy, regular use of sedative drugs, drug or alcohol abuse, and previous active treatment for painful TMD within the last month. Also chronic facial pain (GCPS value 3 or 4), dental pain, systemic pathologies (e.g., rheumatoid arthritis), facial trauma or surgery, neuropathic pain, and people with insufficient fluency in German.	‐ **Group A:** Sensorimotor training using a prefabricated device (RehaBite; Dentrade International, Köln, Germany) with liquid‐filled elastic pads. First patients had to bite it at the maximum force (without inducing or increasing pain) and the device recorded that force. The protocol was: ‐Holding the determined force level for 10 s ‐Slow active opening of the jaw until the pain barrier is reached and additional slight stretching the musculature by means of the thumb and the middle finger ‐Jaw closing, without tooth contact, and resting for 10 s ‐Posology: 10 reps for 3 sets with 30s rest, replicated for 3 times a day.	**Group B:** received a Michigan‐type hard acrylic oral splint to wear during sleep only.	‐**Pain intensity**: rated on NRS scale and characteristic pain intensity (CPI) measured with the GCPS. ‐**The EMG activity of the chewing muscles**: recorded using Ag/AgCl bipolar surface electrodes. **‐Submaximum bite force**: controlled using a specific device (BiteFork; ViMeS, Igel, Germany)	‐**T0**: pain, EMG activity, submaximal bite force ‐**T1** (2 weeks): pain ‐**T2** (6 weeks): pain ‐**T3** (12 weeks): pain, EMG activity, submaximal bite force	‐**Pain**: CPI was reduced between T0 and T3 by 53% for group A (sensorimotor training) and by 40% for group B (splint). At T3 scores at NRS were: 7.5 ± 2.8 for group A vs. 9.6 ± 0.7 for group B. ‐**Neuromuscular performances**: For group A, submaximum activity (of masseter and temporal muscles) changed significantly from T0 to T3. For group B, such changes were not observed. Meanwhile the maximum bite force changed significantly for both groups for the masseter muscle, and, for group A only, for the temporal muscle also.
Barbosa et al. [45] Year: 2019 Country: Brazil	‐**Sample**: 46 women (between 18 and 45 years old) with chronic TMD ‐**TMD type**: myalgia according to RCD/TMD ‐**TMD diagnostic criteria:** The Research Diagnostic Criteria for Temporomandibular Disorders (RDC/TMD‐Axis I) ‐**Inclusion criteria:** a minimum of 28 permanent teeth and age between 18 and 45 years ‐**Exclusion criteria:** history of trauma on the face and on the temporomandibular joint (TMJ), systemic diseases such as arthritis, pain attributable to confirmed migraine, chronic use (more than 6 months) of any analgesic, anti‐inflammatory or psychiatric drugs, acute infection or other significant disease of the teeth, ears, eyes, nose, or throat, and neurological or cognitive deficit.	**‐Group A:** Protocol of resistance exercises (biting endurance), controlled by a visual biofeedback, performed twice a week for 8 weeks (16 sessions). The external load, the repetitions, the rest before contraction and the series were progressively increased during the weeks of training, while the time of contraction, the interval between repetitions and the rest between series were progressively decreased.	**Group B:** Simulated low intensity laser therapy (off mode) applied to the following sites of positioning: the TMJ, the anterior temporal muscle and the masseter muscle, bilaterally	‐**Pain perception**: measured with VAS scores—**PPT** ‐**Time until fatigue during the biting task, muscle efficiency and effects on muscle excitation**: data were extracted using the Miotec SuiteTM Software (MiotecTM, Biomedical Equipments)	Each outcome was evaluated at: ‐**Baseline** **−4 weeks** **−8 weeks**	‐ **Pain:** the intervention group showed lower values of perceived pain scores compared with the placebo group; **‐Neuromuscular performance:** The time until fatigue and the muscle efficiency were higher on the intervention group. No between‐groups differences were observed for the biting force, the PPT and the muscle excitation.
Gavish et al. [46] Year: 2006 Country: Israel	‐**Sample**: 20 females (aged between 20 and 45 years) with myofascial pain without limited opening ‐**TMD type**: myofascial pain (MFP) ‐**TMD diagnostic criteria**: Research Diagnostic Criteria for TMD (RDC/TMD) ‐**Inclusion criteria**: masticatory muscle pain for at least six months, sensitivity to palpation of the masseter muscle at moderate (grade 2) to severe (grade 3) level at the pain side. ‐**Exclusion criteria**: Patients with temporomandibular joint disease or disorder diagnosed clinically or radiologically, systemic chronic disease, continuous use of medication, history of trauma to the facial or cervical regions and previous treatment for the MFP during the previous six months.	**Group A** (Chewing Exercise Protocol): Two units of sugarless chewing gum were chewed three times daily for ten minutes (weeks 1 and 2), increasing to 15 min three times daily (weeks 3 and 4), 20 min three times daily (weeks 5 and 6), and 30 min three times daily (weeks 7 and 8). Patients were instructed to chew at their own rate.	**Group B** (counselling): patients received only support and encouragement.	‐**Present pain** at the time of examination on VAS; **‐Pain intensity** (mean value of present pain, worst pain and average pain during the last month) on VAS—**Pain level at end of chewing phase of the test** (VAS); ‐**EMG measurements of maximal voluntary clench** (of ten sec duration) to assess muscle performance ‐**Muscle sensitivity to palpation** (mean value for masticatory muscles): (0 = no pain, 1—mild pain, 2—moderate pain, 3—severe pain)	The experiment lasted eight weeks, included 5 sessions 2 weeks apart and they performed two evaluations of all the outcomes (at S‐1 and S‐5). Sessions 2–3–4 were to report patients' condition, reassurance, support, and encouragement.	**‐Pain:** There was a decrease in the level of pain parameters (present pain and pain intensity) during the study in both groups, a paired t‐test revealed a statistically significant reduction only for pain intensity in the exercise group. **‐Neuromuscular performance:** EMG during maximal voluntary clench in the exercise group was significantly higher at S‐5 when compared to the EMG at S‐1. While measurements in the control group did not change. The muscle sensitivity to palpation was stable in both groups

With regards to the intervention protocols, the three studies described different dosages of RT, and they all suggested different methods and devices to apply resistance during the exercises. Giannakopoulos et al. [27] utilised sensorimotor training, Barbosa et al. [46] provided visual biofeedback, and Gavish et al. [47] asked the patients to chew a particular chewing gum for a period of time progressively increasing from 10 to 30 min. The resistance devices used across studies varied significantly in terms of texture and ease of use (i.e., sensorimotor device, biofeedback tools and chewing gum). Training protocols also differed markedly: for example, in the study by Giannakopoulos et al. [27], patients were asked to perform their exercises three times every day for 12 weeks, whereas the study by Barbosa et al. [46] used a training frequency of twice per week for 16 sessions (8 weeks), and the study by Gavish et al. [47] instructed patients to perform exercises three times daily for 8 weeks (specifically 5 sessions two weeks apart). The assessment points among the studies were relatively consistent (e.g., short‐term, 8 weeks, 12 weeks), with pain identified as the primary outcome in all trials. However, discrepancies were noted in the pain measurement scales across studies [27, 46, 47]. Giannakopoulos et al. [24] considered the characteristic pain intensity scale (CPI), computed based on the average between NRS values for mean, worst, and current pain over the past 6 months. Barbosa et al. [46] used a visual analogue scale (VAS) of 0–100 mm (0 being no pain and 100 being the worst pain ever experienced) for pain intensity during temporalis palpation with 1 kg of pressure. Lastly, the study by Gavish et al. [47] assessed pain using various methods: current pain on a VAS, average pain intensity over the past month (also on the VAS), the pain relief scale (i.e., measuring reduction in pain on the VAS), and lastly an average value between the pain intensities from palpation on various masticatory muscles.

### 3.3 Risk of Bias and Certainty of Evidence

**FIGURE 2:**
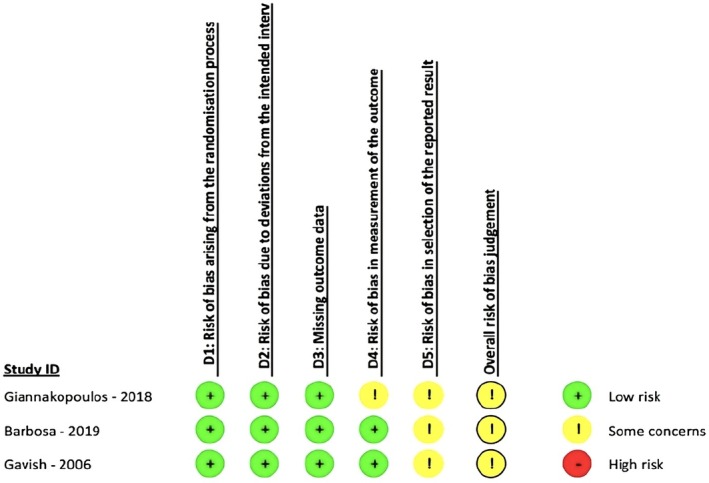
Risk of bias 2 rating assessment for each study in all domains.

The risk of bias assessment results for the included studies are displayed in Figure 2. The included studies [27, 46, 47] raised some concerns regarding the risk of bias. While none had bias related to missing outcome data, all showed moderate bias concerning selective reporting of results. Two studies [27, 47] lacked a registered trial protocol, while one [46] had a protocol but omitted statistical details, raising concerns. Although Barbosa et al. [46] employed randomisation, selection bias may have been introduced as participants were not treatment seekers. The study by Gavish et al. [47] had low bias in randomisation but did not clarify the ‘age‐matched’ groups. However, two studies [46, 47] used blinded assessors, reducing outcome measurement bias.

The certainty of evidence for the included studies, based on the GRADE assessment, was rated as very low, primarily due to serious inconsistency and imprecision. Pain was the only outcome consistently measured across studies, but the variability of the outcome measures used to assess pain intensity and the heterogeneity of comparator interventions led to significant inconsistency. Additionally, the small sample sizes resulted in wide confidence intervals, reducing statistical power and introducing serious imprecision. However, no issues were found with indirectness or publication bias. As a result, the overall quality of evidence for pain intensity was determined to be very low, as outlined in Table 3.

**TABLE 3:** GRADE assessment of studies comparing RT vs. other interventions for reducing pain intensity in patients with TMDs.

Reference and study design	Participants in RT and in comparison groups	Limitations [RoB2]	Inconsistency	Indirectness	Imprecision	Publication bias	Quality of evidence grades
Giannakopoulos et al. [27] 2018 (RCT) Barbosa et al. [45] 2019 (RCT) Gavish et al. [46] 2006 (Pilot study)	RT (*n* = 56) Comparison (*n* = 55)	Unclear^a^	Serious^b^	Not serious	Serious^c^	Undetected	Very low

^a^ Overall risk of bias: three studies ‘Some Concerns’.

^b^ Heterogeneity of comparator.

^c^ Small sample size and unsatisfactory confidence interval.

### 3.4 Results of Individual Studies

#### 3.4.1 Primary Outcomes

All three studies demonstrated a reduction in pain intensity for both the intervention and comparison groups at follow‐up assessments [27, 46, 47]. The study by Giannakopoulos et al. [27] observed a reduction in pain intensity following both the sensorimotor training and the splint therapy, with a significantly larger decrease for the sensorimotor training group at the 12‐week follow‐up. The authors concluded that sensorimotor training could be a cost‐effective alternative compared to splints and used as standard care to reduce pain. However, there was no control over the intervention, as it relied on self‐administered use, particularly during home exercises. According to Barbosa et al. [46], the RT group reported progressively lower pain intensity, and the major between‐group difference was found at the 8‐week follow‐up. However, patients reporting pain provoked by palpation rather than familiar or spontaneous symptoms could represent a limitation of this study. In the trial conducted by Gavish et al. [47], only the intervention group showed a statistically significant reduction for pain intensity (mathematical mean value measured on the VAS describing present pain, worst pain and average pain) and disability score at the final examination (i.e., evaluation at 8 weeks). Moreover, the subjective report of pain relief also showed significant improvement (*p* = 0.019) in the exercise group only. However, as the trial authors suggested, a larger study population, harder material used for the resistance appliance and an extended chewing protocol should be preferred for future studies.

**FIGURE 3:**
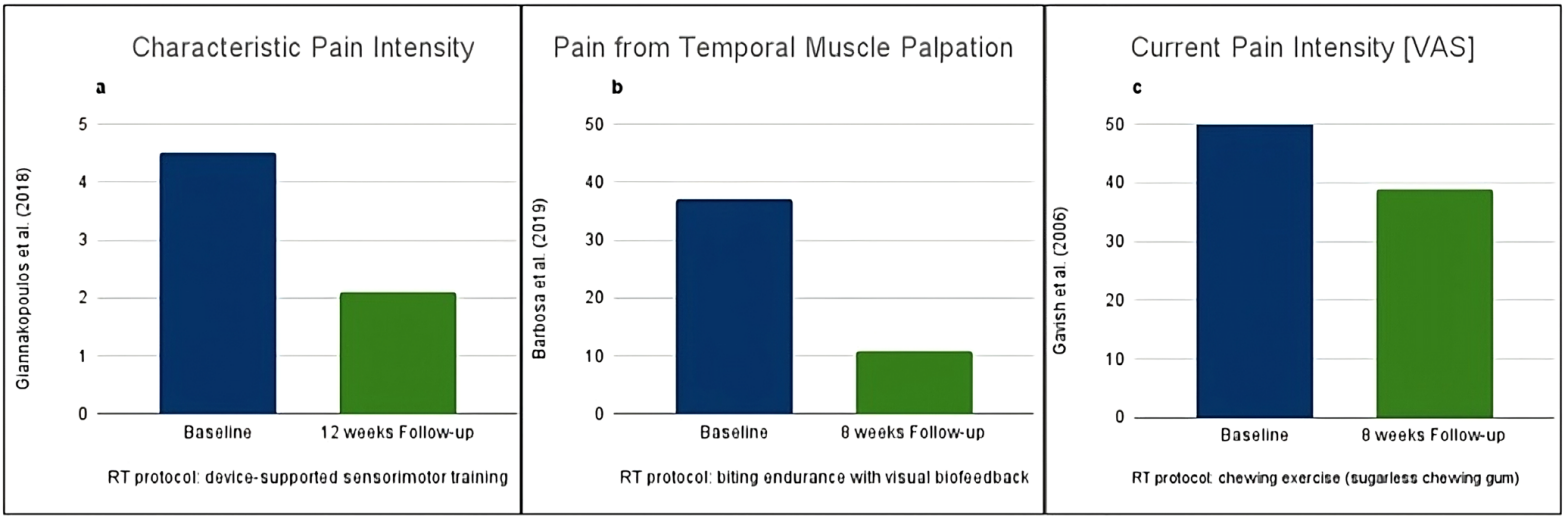
The effect of RT on pain intensity in the included studies, from baseline to follow‐up. (a) Giannakopoulos et al. (2018) reported a significant reduction in pain intensity measured by the characteristic pain intensity scale from baseline to the 12 week follow‐up. The RT protocol involved device‐supported sensorimotor training. (b) Barbosa et al. (2019) observed a significant reduction in temporal muscle pain upon palpation, measured using a VAS, from baseline to 8 weeks. The RT protocol employed biting endurance with visual biofeedback. (c) Gavish et al. (2006) noted a significant reduction in current pain intensity, measured using a VAS, from baseline to 8 weeks. The RT protocol involved progressive chewing exercises using sugarless gum.

Overall, RT was found to be significantly effective in all studies due to the decrease in pain intensity in the intervention groups at the assessment points [27, 46, 47], as shown in Figure 3. Nevertheless, the effect of RT in comparison to other interventions remains unclear due to the small sample sizes of the included studies and heterogeneity in pain outcome measures and comparators.

#### 3.4.2 Secondary Outcomes

Significant enhancements in neuromuscular performance, including masticatory muscle efficiency and MVIC, were consistently observed following RT. Barbosa et al. [46] demonstrated increased muscle efficiency and prolonged time until fatigue at 8 weeks. Similarly, Giannakopoulos et al. [27] and Gavish et al. [47] reported improved MVIC, with Giannakopoulos et al. [27] highlighting notable changes in submaximal masseter and temporalis muscle activity at 12 weeks. However, no significant differences in PPT were identified between groups, and MMO was not evaluated in any of the studies [27, 46, 47].

These findings suggest that RT contributes to notable improvements in neuromuscular performance for patients with TMDs. However, such effects require further exploration in future studies with larger sample sizes and less heterogeneity in the RT protocol.

## 4 Discussion

Systematic reviews have consistently highlighted the effectiveness of physical therapy for the management of patients with TMDs, supporting a conservative, evidence‐based approach aligned with treatment guidelines, which emphasise behavioural modifications alongside physical rehabilitation [12, 13, 14, 15, 16, 17, 30, 48, 49]. Exercise, specifically, has been shown to counteract chronic low‐grade systemic inflammation, enhance muscle health and modulate inflammatory pathways by promoting anti‐inflammatory factors and reducing inflammatory markers [24, 25, 50]. RT in particular, is well established for enhancing load tolerance, facilitating tissue adaptation and driving physiological changes beneficial across various musculoskeletal conditions, including low back pain, neck pain, knee osteoarthritis and tendinopathies [19, 20, 21, 22, 23, 51]. By improving muscle strength, reducing pain and enhancing functional outcomes, RT confers substantial benefits to patients with these conditions [19, 20, 21, 22, 23, 51]. In patients with TMDs, neuromuscular performance of the masticatory muscles is frequently impaired, resulting in reduced bite force, diminished force control and compromised jaw coordination [8, 52]. Thus, RT may offer therapeutic benefits by enhancing neuromuscular function in patients with TMD [27, 46, 47]. Furthermore, emerging evidence indicates that awake bruxism (i.e., jaw bracing) is notably more frequent among individuals with TMD [28, 53] and that TMD‐related pain may be exacerbated by overload of the masticatory muscles, contributing to muscle and joint discomfort [53]. RT, by improving load tolerance, may therefore hold promise in managing TMD symptoms associated with musculoskeletal overload, including those stemming from bruxism [24, 28, 29, 45].

Previous systematic reviews have investigated the effects of exercise on TMD, but the findings have been inconsistent, likely due to the varied exercise interventions evaluated [17, 18, 30]. The current systematic review supports findings from studies reporting improvements in neuromuscular performance, such as bite force, following exercise interventions for patients with TMD [18, 33], and aligns with research noting exercise‐related pain reduction [17, 31]. It also concurs with a review [32] that observed no definitive advantage of exercise over splint therapy in reducing pain at a follow‐up. Notably, all studies included in this systematic review reported significant pain reduction during follow‐up assessments at the end of the RT intervention [27, 46, 47]. One study [46] also highlighted that targeted muscle endurance training decreased pain and enhanced muscle efficiency by increasing fatigue tolerance and further found RT to be superior to placebo in reducing pain and improving neuromuscular efficiency. Although RT outperformed sham therapy or counselling alone [46, 47], it did not exhibit clear superiority over splint therapy [27]. Interestingly, a reduction of pain intensity by 53% was observed following sensorimotor training, whereas a reduction by only 40% was observed for the group using splint therapy; yet patients stated that they preferred splint therapy as it is a passive treatment and easier to use, leading to two dropouts in the RT group. Conversely, another study [47] reported high adherence to the exercise protocol.

Although the findings from this systematic review support the effectiveness of RT in reducing pain and improving neuromuscular performance in TMDs, these findings should be interpreted with caution due to several limitations. The restrictive inclusion criteria, focusing solely on RT without combined treatments, limited the number of studies and resulted in a small sample size of 108 participants. Given the high prevalence of TMDs [1, 2, 4], this highlights the scarcity of research in this area. Additionally, the studies exclusively included women, limiting the generalisability of the findings. All studies [27, 46, 47] focused on myogenic TMD, with one specifically targeting chronic myofascial pain [47], and none included patients with arthralgia, preventing subgroup analyses by TMD type or sex. The risk of bias assessment indicated ‘some concerns’ across all studies, suggesting methodological weaknesses that may impact reliability. The studies also exhibited significant heterogeneity, including differences in comparators, outcome measures for pain and neuromuscular performance and RT protocols. This diversity in outcome measures made it difficult to draw definitive conclusions about the effectiveness of RT. Furthermore, substantial differences in RT interventions, such as resistance devices and training protocols, further precluded comparisons. These methodological variations challenge the evaluation of the overall impact of RT. Indeed, the significant heterogeneity prevented meta‐analysis, and, combined with the moderate risk of bias and small sample sizes, resulted in very low‐level evidence.

Although the conclusion of this systematic review is based on low‐level evidence, it effectively highlights key areas for future research. Addressing these gaps will enhance scientific rigour and provide better guidance for clinical practice, ultimately improving care for patients with TMDs. Future trials should focus on isolating exercise interventions, avoiding combinations with other treatments or mixing various exercise types in order to fully appreciate the benefit of single types of exercises/interventions. Additionally, a full description of the exercise protocol, including dosage and frequency, is crucial for generating precise evidence. This approach would enhance internal validity and provide a clearer understanding of the specific effects of exercise programmes on patient outcomes. Researchers should account for the variability within TMD populations by distinguishing specific subtypes and designing exercise protocols tailored to each condition. Furthermore, given the importance of MMO in evaluating disability among TMD patients, future studies evaluating the effectiveness on RT should include this outcome in their assessments. Adherence and compliance are critical factors in physical therapy, especially for active exercise performed outside supervised settings. Given that there is typically lower adherence to exercise‐based interventions compared to passive treatments, studies must incorporate compliance assessments. This approach will provide a clearer understanding of patient engagement and its influence on treatment outcomes. Lastly, to strengthen the robustness and reliability of findings, studies must provide a detailed description of study protocols and statistical analyses. It is essential to register trials in advance with clear declarations of intended statistical methods; this is vital to reduce the risk of bias and enhance the credibility of results. Without such transparency, the risk of bias increases by limiting confidence in findings.

In conclusion, RT is beneficial for reducing pain and improving neuromuscular performance in patients with TMDs, albeit with very low quality of evidence. Future research should focus on testing isolated exercise interventions, standardising exercise protocols and incorporating adherence assessments. Additionally, future studies should include larger samples encompassing both sexes and all TMD subtypes. Addressing these gaps will help establish stronger evidence for the role of RT in TMD management.

## Author Contributions

All authors fully contributed to the manuscript. G.A. and D.F. were responsible for the initial conception of the review. All authors contributed to the final study conception. G.A. and G.P. were the two reviewers at each stage supported by D.F. in case of disagreement. P.B. and E.B. contributed to the Introduction, particularly regarding the potential impact of bruxism overload on TMDs and effects of resistance training. C.B. and A.M. assisted in interpreting results and contributed to the Results section (also developing figures and tables). M.P. and A.G. contributed to the Discussion, focusing on future research directions. F.M. and F.M. critically revised the manuscript, enhancing sections on limitations of the review, results interpretation and clinical implications. All authors have contributed to study conception, methodology, data extraction and analysis, manuscript writing, and critical revisions, data interpretation, conclusions and dissemination. All authors have read, contributed to and agreed upon the final manuscript. G.A. is the guarantor.

## Conflicts of Interest

The authors declare no conflicts of interest.

## Peer Review

The peer review history for this article is available at https://www.webofscience.com/api/gateway/wos/peer‐review/10.1111/joor.14021.

## Supporting information


Data S1.

Data S2.

Data S3.

Data S4.

## Data Availability

The data supporting the findings of this study are available within the article and its Supporting Materials.
