# Supplementary material for: Effectiveness of Resistance Training of Masticatory Muscles for Patients With Temporomandibular Disorders: A Systematic Review
Source: J Oral Rehabil. 2025 May 25;52(9):1505–17. doi: 10.1111/joor.14021 (PMC12408958; doi:10.1111/joor.14021)
Supplement: Supplementary file 2 — Data S2. [file JOOR-52-1505-s004.docx]

**Additional file 2- Search strategies**

EFFECTIVENESS OF RESISTANCE TRAINING OF MASTICATORY MUSCLES FOR PATIENTS WITH TEMPOROMANDIBULAR DISORDERS: A SYSTEMATIC REVIEW

**PUBMED (1082 results)**

("temporal muscle"[MeSH Terms] OR "masseter muscle"[MeSH Terms] OR "masticatory muscles"[MeSH Terms] OR "FACIAL PAIN"[All Fields] OR "FACIAL PAIN"[MeSH Terms] OR "temporomandibular joint dysfunction syndrome"[MeSH Terms] OR "craniomandibular disorders"[MeSH Terms] OR "temporomandibular joint"[MeSH Terms] OR "temporomandibular joint disorders"[MeSH Terms] OR "craniomandibul*"[All Fields] OR "temporomandibul*"[All Fields] OR "Temporomandibular pain"[All Fields] OR "Jaw dysfunction"[All Fields] OR "Jaw pain"[All Fields] OR "craniomandibular dysfunction*"[All Fields] OR "Craniomandibular pain"[All Fields] OR "Orofacial Pain"[All Fields] OR "temporal muscle*"[All Fields] OR "masseter muscle*"[All Fields] OR "TMJ"[All Fields] OR "TMD"[All Fields] OR "Temporomandibular Dysfunction"[All Fields] OR "Temporomandibular Joint Dysfunction"[All Fields] OR "Temporomandibular joint syndrome"[All Fields] OR "craniomandibular disorder*"[All Fields] OR "temporomandibular joint*"[All Fields] OR "temporomandibular joint disorder*"[All Fields] OR "temporomandibular disorder*"[All Fields]) AND ("orthopedic*"[All Fields] OR "orthopedics"[MeSH Terms] OR "orthopaedic*"[All Fields] OR "musculoskeletal therap*"[All Fields] OR "physical therapy modalities"[MeSH Terms] OR "physical therapy modalities"[MeSH Terms] OR "exercise therapy"[MeSH Terms] OR "resistance training"[MeSH Terms] OR "exercise movement techniques"[MeSH Terms] OR "physical therap*"[All Fields] OR "physiotherap*"[All Fields] OR ("exercise"[MeSH Terms] OR "exercise"[All Fields] OR "exercises"[All Fields] OR "exercise therapy"[MeSH Terms] OR ("exercise"[All Fields] AND "therapy"[All Fields]) OR "exercise therapy"[All Fields] OR "exercise s"[All Fields] OR "exercised"[All Fields] OR "exerciser"[All Fields] OR "exercisers"[All Fields] OR "exercising"[All Fields]) OR "mouth rehabilitation"[MeSH Terms] OR ("rehabilitant"[All Fields] OR "rehabilitants"[All Fields] OR "rehabilitate"[All Fields] OR "rehabilitated"[All Fields] OR "rehabilitates"[All Fields] OR "rehabilitating"[All Fields] OR "rehabilitation"[MeSH Terms] OR "rehabilitation"[All Fields] OR "rehabilitations"[All Fields] OR "rehabilitative"[All Fields] OR "rehabilitation"[MeSH Subheading] OR "rehabilitation s"[All Fields] OR "rehabilitational"[All Fields] OR "rehabilitator"[All Fields] OR "rehabilitators"[All Fields]) OR "rehabilitation"[MeSH Terms]) AND ("trial*"[All Fields] OR "clinical trials as topic"[MeSH Terms] OR "placebo effect"[MeSH Terms])

**MEDLINE (ovid interface) (1253 results)**

(exp "temporal muscle"/ OR exp "masseter muscle"/ OR exp "masticatory muscles"/ OR "FACIAL PAIN".af. OR exp "FACIAL PAIN"/ OR exp "temporomandibular joint dysfunction syndrome"/ OR exp "craniomandibular disorders"/ OR exp "temporomandibular joint"/ OR exp "temporomandibular joint disorders"/ OR craniomandibul*.af. OR temporomandibul*.af. OR "Temporomandibular pain".af. OR "Jaw dysfunction".af. OR "Jaw pain".af. OR "craniomandibular dysfunction*".af. OR "Craniomandibular pain".af. OR "Orofacial Pain".af. OR "temporal muscle*".af. OR "masseter muscle*".af. OR TMJ.af. OR TMD.af. OR "Temporomandibular Dysfunction".af. OR "Temporomandibular Joint Dysfunction".af. OR "Temporomandibular joint syndrome".af. OR "craniomandibular disorder*".af. OR "temporomandibular joint*".af. OR "temporomandibular joint disorder*".af. OR "temporomandibular disorder*".af.) AND (orthopedic*.af. OR exp orthopedics/ OR orthopaedic*.af. OR "musculoskeletal therap*".af. OR exp "physical therapy modalities"/ OR exp "physical therapy modalities"/ OR exp "exercise therapy"/ OR exp "resistance training"/ OR exp "exercise movement techniques"/ OR "physical therap*".af. OR physiotherap*.af. OR (exp exercise/ OR exercise.af. OR exercises.af. OR exp "exercise therapy"/ OR (exercise.af. AND therapy.af.) OR "exercise therapy".af. OR "exercise s".af. OR exercised.af. OR exerciser.af. OR exercisers.af. OR exercising.af.) OR exp "mouth rehabilitation"/ OR (rehabilitant.af. OR rehabilitants.af. OR rehabilitate.af. OR rehabilitated.af. OR rehabilitates.af. OR rehabilitating.af. OR exp rehabilitation/ OR rehabilitation.af. OR rehabilitations.af. OR rehabilitative.af. OR rehabilitation.fs. OR "rehabilitation s".af. OR rehabilitational.af. OR rehabilitator.af. OR rehabilitators.af.) OR exp rehabilitation/) AND (trial*.af. OR exp "clinical trials as topic"/ OR exp "placebo effect"/)

**EMBASE (ovid interface)**

(exp "temporal muscle"/ OR exp "masseter muscle"/ OR exp "masticatory muscles"/ OR "FACIAL PAIN".af. OR exp "FACIAL PAIN"/ OR exp "temporomandibular joint dysfunction syndrome"/ OR exp "craniomandibular disorders"/ OR exp "temporomandibular joint"/ OR exp "temporomandibular joint disorders"/ OR craniomandibul*.af. OR temporomandibul*.af. OR "Temporomandibular pain".af. OR "Jaw dysfunction".af. OR "Jaw pain".af. OR "craniomandibular dysfunction*".af. OR "Craniomandibular pain".af. OR "Orofacial Pain".af. OR "temporal muscle*".af. OR "masseter muscle*".af. OR TMJ.af. OR TMD.af. OR "Temporomandibular Dysfunction".af. OR "Temporomandibular Joint Dysfunction".af. OR "Temporomandibular joint syndrome".af. OR "craniomandibular disorder*".af. OR "temporomandibular joint*".af. OR "temporomandibular joint disorder*".af. OR "temporomandibular disorder*".af.) AND (orthopedic*.af. OR exp orthopedics/ OR orthopaedic*.af. OR "musculoskeletal therap*".af. OR exp "physical therapy modalities"/ OR exp "physical therapy modalities"/ OR exp "exercise therapy"/ OR exp "resistance training"/ OR exp "exercise movement techniques"/ OR "physical therap*".af. OR physiotherap*.af. OR (exp exercise/ OR exercise.af. OR exercises.af. OR exp "exercise therapy"/ OR (exercise.af. AND therapy.af.) OR "exercise therapy".af. OR "exercise s".af. OR exercised.af. OR exerciser.af. OR exercisers.af. OR exercising.af.) OR exp "mouth rehabilitation"/ OR (rehabilitant.af. OR rehabilitants.af. OR rehabilitate.af. OR rehabilitated.af. OR rehabilitates.af. OR rehabilitating.af. OR exp rehabilitation/ OR rehabilitation.af. OR rehabilitations.af. OR rehabilitative.af. OR rehabilitation.fs. OR "rehabilitation s".af. OR rehabilitational.af. OR rehabilitator.af. OR rehabilitators.af.) OR exp rehabilitation/) AND (trial*.af. OR exp "clinical trials as topic"/ OR exp "placebo effect"/)

**SCOPUS (838 results)**

( ( TITLE-ABS-KEY ( "temporal muscle" ) OR TITLE-ABS-KEY ( "masseter muscle" ) OR TITLE-ABS-KEY ( "masticatory muscles" ) OR TITLE-ABS-KEY ( "FACIAL PAIN" ) OR TITLE-ABS-KEY ( "FACIAL PAIN" ) OR TITLE-ABS-KEY ( "temporomandibular joint dysfunction syndrome" ) OR TITLE-ABS-KEY ( "craniomandibular disorders" ) OR TITLE-ABS-KEY ( "temporomandibular joint" ) OR TITLE-ABS-KEY ( "temporomandibular joint disorders" ) OR TITLE-ABS-KEY ( craniomandibul* ) OR TITLE-ABS-KEY ( temporomandibul* ) OR TITLE-ABS-KEY ( "Temporomandibular pain" ) OR TITLE-ABS-KEY ( "Jaw dysfunction" ) OR TITLE-ABS-KEY ( "Jaw pain" ) OR TITLE-ABS-KEY ( "craniomandibular dysfunction*" ) OR TITLE-ABS-KEY ( "Craniomandibular pain" ) OR TITLE-ABS-KEY ( "Orofacial Pain" ) OR TITLE-ABS-KEY ( "temporal muscle*" ) OR TITLE-ABS-KEY ( "masseter muscle*" ) OR TITLE-ABS-KEY ( tmj ) OR TITLE-ABS-KEY ( tmd ) OR TITLE-ABS-KEY ( "Temporomandibular Dysfunction" ) OR TITLE-ABS-KEY ( "Temporomandibular Joint Dysfunction" ) OR TITLE-ABS-KEY ( "Temporomandibular joint syndrome" ) OR TITLE-ABS-KEY ( "craniomandibular disorder*" ) OR TITLE-ABS-KEY ( "temporomandibular joint*" ) OR TITLE-ABS-KEY ( "temporomandibular joint disorder*" ) OR TITLE-ABS-KEY ( "temporomandibular disorder*" ) ) AND ( TITLE-ABS-KEY ( orthopedic* ) OR TITLE-ABS-KEY ( orthopedics ) OR TITLE-ABS-KEY ( orthopaedic* ) OR TITLE-ABS-KEY ( "musculoskeletal therap*" ) OR TITLE-ABS-KEY ( "physical therapy modalities" ) OR TITLE-ABS-KEY ( "physical therapy modalities" ) OR TITLE-ABS-KEY ( "exercise therapy" ) OR TITLE-ABS-KEY ( "resistance training" ) OR TITLE-ABS-KEY ( "exercise movement techniques" ) OR TITLE-ABS-KEY ( "physical therap*" ) OR TITLE-ABS-KEY ( physiotherap* ) OR ( TITLE-ABS-KEY ( exercise ) OR TITLE-ABS-KEY ( exercise ) OR TITLE-ABS-KEY ( exercises ) OR TITLE-ABS-KEY ( "exercise therapy" ) OR ( TITLE-ABS-KEY ( exercise ) AND TITLE-ABS-KEY ( therapy ) ) OR TITLE-ABS-KEY ( "exercise therapy" ) OR TITLE-ABS-KEY ( "exercise s" ) OR TITLE-ABS-KEY ( exercised ) OR TITLE-ABS-KEY ( exerciser ) OR TITLE-ABS-KEY ( exercisers ) OR TITLE-ABS-KEY ( exercising ) ) OR TITLE-ABS-KEY ( "mouth rehabilitation" ) OR ( TITLE-ABS-KEY ( rehabilitant ) OR TITLE-ABS-KEY ( rehabilitants ) OR TITLE-ABS-KEY ( rehabilitate ) OR TITLE-ABS-KEY ( rehabilitated ) OR TITLE-ABS-KEY ( rehabilitates ) OR TITLE-ABS-KEY ( rehabilitating ) OR TITLE-ABS-KEY ( rehabilitation ) OR TITLE-ABS-KEY ( rehabilitation ) OR TITLE-ABS-KEY ( rehabilitations ) OR TITLE-ABS-KEY ( rehabilitative ) OR TITLE-ABS-KEY ( rehabilitation ) OR TITLE-ABS-KEY ( "rehabilitation s" ) OR TITLE-ABS-KEY ( rehabilitational ) OR TITLE-ABS-KEY ( rehabilitator ) OR TITLE-ABS-KEY ( rehabilitators ) ) OR TITLE-ABS-KEY ( rehabilitation ) ) AND ( TITLE-ABS-KEY ( trial* ) OR TITLE-ABS-KEY ( "clinical trials as topic" ) OR TITLE-ABS-KEY ( "placebo effect" ) ) )

**COCHRANE CENTRAL REGISTER OF CONTROLLED TRIALS (366 results: 360 trials and 6 reviews)**

(("temporal muscle" OR "masseter muscle" OR "masticatory muscles" OR "FACIAL PAIN" OR "FACIAL PAIN" OR "temporomandibular joint dysfunction syndrome" OR "craniomandibular disorders" OR "temporomandibular joint" OR "temporomandibular joint disorders" OR craniomandibul* OR temporomandibul* OR "Temporomandibular pain" OR "Jaw dysfunction" OR "Jaw pain" OR "craniomandibular dysfunction*" OR "Craniomandibular pain" OR "Orofacial Pain" OR "temporal muscle*" OR "masseter muscle*" OR TMJ OR TMD OR "Temporomandibular Dysfunction" OR "Temporomandibular Joint Dysfunction" OR "Temporomandibular joint syndrome" OR "craniomandibular disorder*" OR "temporomandibular joint*" OR "temporomandibular joint disorder*" OR "temporomandibular disorder*") AND ("orthopedic*" OR orthopedics OR "orthopaedic*" OR "musculoskeletal therap*" OR "physical therapy modalities" OR "physical therapy modalities" OR "exercise therapy" OR "resistance training" OR "exercise movement techniques" OR "physical therap*" OR "physiotherap*" OR (exercise OR exercises OR "exercise therapy" OR (exercise AND therapy) OR "exercise therapy" OR "exercise s" OR exercised OR exerciser OR exercisers OR exercising) OR "mouth rehabilitation" OR (rehabilitant OR rehabilitants OR rehabilitate OR rehabilitated OR rehabilitates OR rehabilitating OR rehabilitation OR rehabilitations OR rehabilitative OR rehabilitation OR "rehabilitation s" OR rehabilitational OR rehabilitator OR rehabilitators) OR rehabilitation) AND (trial* OR "clinical trials as topic" OR "placebo effect"))

**WEB OF SCIENCE (1125 results)**

(ALL="temporal muscle" OR ALL="masseter muscle" OR ALL="masticatory muscles" OR ALL="FACIAL PAIN" OR ALL="FACIAL PAIN" OR ALL="temporomandibular joint dysfunction syndrome" OR ALL="craniomandibular disorders" OR ALL="temporomandibular joint" OR ALL="temporomandibular joint disorders" OR ALL=craniomandibul* OR ALL=temporomandibul* OR ALL="Temporomandibular pain" OR ALL="Jaw dysfunction" OR ALL="Jaw pain" OR ALL="craniomandibular dysfunction*" OR ALL="Craniomandibular pain" OR ALL="Orofacial Pain" OR ALL="temporal muscle*" OR ALL="masseter muscle*" OR ALL=TMJ OR ALL=TMD OR ALL="Temporomandibular Dysfunction" OR ALL="Temporomandibular Joint Dysfunction" OR ALL="Temporomandibular joint syndrome" OR ALL="craniomandibular disorder*" OR ALL="temporomandibular joint*" OR ALL="temporomandibular joint disorder*" OR ALL="temporomandibular disorder*") AND (ALL=orthopedic* OR ALL=orthopedics OR ALL=orthopaedic* OR ALL="musculoskeletal therap*" OR ALL="physical therapy modalities" OR ALL="physical therapy modalities" OR ALL="exercise therapy" OR ALL="resistance training" OR ALL="exercise movement techniques" OR ALL="physical therap*" OR ALL=physiotherap* OR (ALL=exercise OR ALL=exercise OR ALL=exercises OR ALL="exercise therapy" OR (ALL=exercise AND ALL=therapy) OR ALL="exercise therapy" OR ALL="exercise s" OR ALL=exercised OR ALL=exerciser OR ALL=exercisers OR ALL=exercising) OR ALL="mouth rehabilitation" OR (ALL=rehabilitant OR ALL=rehabilitant OR ALL=rehabilitate OR ALL=rehabilitated OR ALL=rehabilitated OR ALL=rehabilitating OR ALL=rehabilitation OR ALL=rehabilitation OR ALL=rehabilitations OR ALL=rehabilitative OR ALL=rehabilitation OR ALL="rehabilitation s" OR ALL=rehabilitationa OR ALL=rehabilitator OR ALL=rehabilitator) OR ALL=rehabilitation) AND (ALL="Randomised controlled trial" OR ALL="randomized controlled trial*" OR ALL=trial* OR ALL=random* OR ALL="randomized controlled trials as topic" OR ALL="random allocation" OR ALL="clinical trials as topic" OR ALL="placebo effect")
