# Supplementary material for: Effectiveness of Resistance Training of Masticatory Muscles for Patients With Temporomandibular Disorders: A Systematic Review
Source: J Oral Rehabil. 2025 May 25;52(9):1505–17. doi: 10.1111/joor.14021 (PMC12408958; doi:10.1111/joor.14021)
Supplement: Supplementary file 3 — Data S3. [file JOOR-52-1505-s003.docx]

**Additional file 3 - Inclusion/Exclusion Reviewers Agreement**

EFFECTIVENESS OF RESISTANCE TRAINING OF MASTICATORY MUSCLES FOR PATIENTS WITH TEMPOROMANDIBULAR DISORDERS: A SYSTEMATIC REVIEW

| Phase I – screening titles and abstract | GA | GP | After discussion |
| --- | --- | --- | --- |
| Included | 0 | 0 | N/A |
| Excluded | 2141 | 2138 | 2152 |
| Maybe/Unsure | 36 | 39 | 25 |

| Phase II – screening full texts | GA | GP | After discussion |
| --- | --- | --- | --- |
| Included | 3 | 3 | 3 |
| Excluded | 21 | 20 | 22 |
| Maybe/Unsure | 1 | 2 | 0 |
