# Supplementary material for: Effectiveness of Resistance Training of Masticatory Muscles for Patients With Temporomandibular Disorders: A Systematic Review
Source: J Oral Rehabil. 2025 May 25;52(9):1505–17. doi: 10.1111/joor.14021 (PMC12408958; doi:10.1111/joor.14021)
Supplement: Supplementary file 4 — Data S4. [file JOOR-52-1505-s001.docx]

**Additional file 4 – Agreement of reviewers on Risk of Bias Assessment**

EFFECTIVENESS OF RESISTANCE TRAINING OF MASTICATORY MUSCLES FOR PATIENTS WITH TEMPOROMANDIBULAR DISORDERS: A SYSTEMATIC REVIEW

|  | Overall Risk of Bias - GA | Overall Risk of Bias - GP | Overall Risk of Bias - After discussion |
| --- | --- | --- | --- |
| Barbosa et al. | SOME CONCERN | SOME CONCERN | SOME CONCERN |
| Grace et al. | SOME CONCERN | SOME CONCERN | SOME CONCERN |
| Giannakopoulus et al. | SOME CONCERN | SOME CONCERN | SOME CONCERN |
